# Directing Attention Through Gaze Hints Improves Task Solving in Human–Humanoid Interaction

**DOI:** 10.1007/s12369-018-0473-8

**Published:** 2018-04-06

**Authors:** Eunice Mwangi, Emilia I. Barakova, Marta Díaz-Boladeras, Andreu Català Mallofré, Matthias Rauterberg

**Affiliations:** 1 Department of Industrial Design, Eindhoven University of Technology, Eindhoven, Netherlands; 2 Technical Research Centre for Dependency Care and Autonomous Living (CETpD), Universitat Politècnica de Catalunya, Vilanova i la Geltrú, Barcelona, Spain

**Keywords:** Gaze-based interactions, Gaze perception, Game-based human–robot interaction, Embodied cues, Attentional cues, Directed attention, Facial orientation

## Abstract

In this paper, we report an experimental study designed to examine how participants perceive and interpret social hints from gaze exhibited by either a robot or a human tutor when carrying out a matching task. The underlying notion is that knowing where an agent is looking at provides cues that can direct attention to an object of interest during the activity. In this regard, we asked human participants to play a card matching game in the presence of either a human or a robotic tutor under two conditions. In one case, the tutor gave hints to help the participant find the matching cards by gazing toward the correct match, in the other case, the tutor only looked at the participants and did not give them any help. The performance was measured based on the time and the number of tries taken to complete the game. Results show that gaze hints (helping tutor) made the matching task significantly easier (fewer tries) with the robot tutor. Furthermore, we found out that the robots’ gaze hints were recognized significantly more often than the human tutor gaze hints, and consequently, the participants performed significantly better with the robot tutor. The reported study provides new findings towards the use of non-verbal gaze hints in human–robot interaction, and lays out new design implications, especially for robot-based educative interventions.

## Introduction

At present, robots are showing increasing potential to be incorporated efficiently into various social settings, for example, in educational and therapeutic facilities for children and nursing homes for elderly among others [5, 8, 24]. Accordingly, it is increasingly important to design natural social behaviors for robots. In human–human interactions, people rely on non-verbal cues such as gaze, gestures, body language, and facial expressions to communicate. Feldman and Rimé [17], showed that non-verbal cues contribute significantly to the meaning exchanged in the interaction. Therefore, implementing these cues in social human–robot interaction may increase its naturalness and effectiveness.

**Fig. 1:**
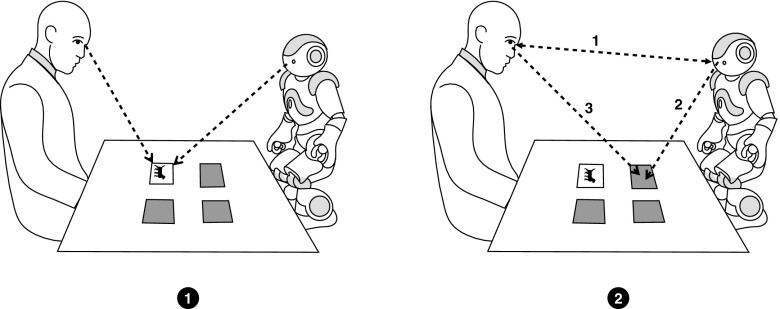
The interaction flow: *P* participant (left) turns over a card; *T* tutor (right) looks at the selected card; tutor gazes at participant to draw attention; *T*—tutor moves on to look at a matching

In an attempt to reach natural interaction, much research has been done into social human–robot interaction on mapping non-verbal human behavior to robots [9, 20]. Among non-verbal behaviors, the gaze is considered as primary source of information [4, 21, 23]. Gaze plays a significant role in social interaction, particularly in directing attention. Moreover, gaze behavior facilitates a range of social functions during human–human interactions such as communicating their emotions, intentions, and what they are attending to [18, 21, 36]. Additionally, it was shown that at a very young age, children can follow the gaze of their parents or their caregivers [10, 11]. On the contrary, children with development disorders such as children with ASD show typical difficulties in producing and reading nonverbal communicative behaviours both gestures and gaze-based [19]. Perhaps, one of the most significant roles of gaze is its capability to direct attention to objects of interest in the environment facilitating the formation of joint visual attention [16, 18]. Joint attention is argued to be the basis for early language learning and normative development in children [6, 10, 11].

Accordingly, the use of eye gaze behaviour in human–robot interaction is gaining the much-needed attention as well. Prior research has demonstrated how gaze can be used to build better interactions with robots [2, 12, 20, 23, 26–28, 30, 35]. “Leakage cues”—that are unintentional and unconscious both in their production and perception have been studied by Mutlu et al. [28] who showed that participants could read and interpret such cues from robots’ gaze. They evaluated the perception of gaze cues that were implemented on Robovie and Geminoid robots. While the above work focuses on unintentional and unconscious influence of gaze cues in a competitive setting, in the current study we focus on deliberate attention directing eye gaze cues in a collaborative setting with a tutor. Palinko et al. [31] studied the impact of the use of eye- or head-based gaze estimation in a human–robot interaction experiment with the iCub robot. The robots used in these studies are able move their eyes independently of the head. Since cheaper and better accessible robots are the more feasible choice for use in education and elderly care, in the current study, we investigate whether robots such as NAO can also convey such deliberate meaning through gaze cues. Differently from [28, 31] we use synchronized simultaneous observations of both the robot and the human’s behavior to reveal precise patterns of gazing behavior. We further examine whether humans can read such cues and accept help from the robot, and, in turn, if these cues influence the decision-making of the interacting human.

For this purpose, we designed an experimental study to evaluate the effects of gaze hints in the context of the educational gameplay. We asked participants to play a matching card game in the presence of a human or a robotic tutor. The aim of the study was to determine if gaze hints from the tutor can direct attention and, consequently influence the choices of human partners. Figure 1 describes the interaction flow for the designed study:

We expected that the participants would notice the gaze of the tutor while the tutor was looking at different cards on the board and that they would follow tutors’ lead to the matching card. Thus, we hypothesized that gaze cues (facial orientation and gaze direction) from the tutor would help in drawing the participants’ attention to the matching card, and subsequently influence their choices. To create a more accurate measure of the interactive behaviors of the human participants, we incorporated eye tracking to make a precise recording of the gaze behavior of the interacting human.

In Sect. 2, we describe prior work on gaze in social human–robot interactions. We further describe the methodology and the design of the human–human and human–robot experimental set-ups in Sect. 3, and the results of the study in Sect. 4. Finally, in Sect. 5, we discuss the findings and limitations of our work and give directions for future work.

## Related Work

Given the critical role of gaze in human communication, research into designing social gaze behaviors for robots has been extensive [2, 12, 15, 31, 35]. Andrist et al.[3] combined three functionalities including face-tracking, head detection, and gaze aversions to create social gaze behaviors for conversational robots. In an evaluation study, the participants indicated they perceived the designed gaze as more intentional. Admoni et al. [1] addressed the impact of frequency and duration of gaze on the perception of attention during the human–robot interaction concluding that shorter, more frequent fixations are better for signifying attention than longer and less frequent fixations.

In a storytelling setting, Mutlu et al. [27] showed that participants recalled the story better when the robot looked longer at them. Yoshikawa et al. [37] explored both responsive and non-responsive gaze cues and found that the responsive gazes gave a strong effect of “feeling of being looked at” during the interaction. Moon et al. [26] studied the effects of gaze behaviors in a handover task. They found that gaze cues can improve the hand-over timing and the subjective experience in hand-over tasks. Boucher et al. [7] studied gaze effects on the speed of communication in both human–human and human–robot interaction collaborative works. Their results demonstrate that human participants can use gaze cues of a human or a robot partner to improve their performance in physical interaction tasks.

Several studies have considered the ability of people to read cues from robot gaze. For example, using a guessing game, Mutlu et al. [28] show that participants can read and interpret leakage cues from robots’ gaze even faster when the robot is more human-like. Their designed gaze behaviors were evaluated on Robovie and Geminoid robotic platforms that can move their eyes independently of the head direction. This poses the question to what degree simpler and more available robots can also perform gaze cuing effectively.

In this line of research, Cuijpers et al. [13] used NAO robot, which has no moveable eyes and measured the region of eye contact with the robot, they concluded that perception of gaze direction with NAO robot is similar to a human looker. Mwangi et al. [29] examined the ability of people to correctly guess the head direction towards different target positions (cards) on a table using the NAO robot. Findings showed that participants perceive the head (gaze) direction of NAO robot more accurately for close objects and also the participants recognized the cards positions left and right of the robot with different accuracy. These related works suggest that robots without movable eyes as NAO robot can be used as well for providing gaze cues.

Prior work has also focused on the role of gaze in joint attention. Pfeiffer-Lessmann et al. [33], examined the timing of gaze patterns in interactions between humans and a virtual human to build a joint operational model for artificial agents. Yu et al. [38] studied the timing patterns of gaze when interacting with either a robot or a human in a word learning task. Their eye-tracking result revealed that people pay more attention to the face region of the robot than that of the human during a word learning task.

In the present comparison study, the main aim is to determine whether gaze hints from a tutor (either human or robot) can direct player attention and therefore influence the choices of human partners in a game-play. In this regard, we devised the following sub-questions: (1) are the provided gaze cues noticed, (2) are they understood as helping behavior and (3) does the help provided by the tutor influence performance. The underlying assumption is that gaze hints can help to cue attention and influence decisions and thoughts, and, therefore, improve the performance.

## Methodology

### The Experimental Setup

To test our hypothesis, we formulated an experimental task, in which participants interacted with a tutor—either a robot or a human—in a variant of the Memory card game. The game is played with fourteen cards (7 matching pairs), with images of black dogs, varying slightly in shape. The choice of the dogs being the same color and not much varying in shape was made to increase the level of difficulty of the game, since the number of pairs is relatively low. On the table, there were 14 cards arranged in a rectangular layout. The layout had six columns and three rows for a total of 18 cards, or 9 pairs. We placed the cards in the first two rows, and two cards in the middle positions of the third row. The distance between the cards on the X-axis was 6 cm; on the Y-axis, it was 10 cm. This arrangement was informed by our prior experiment [29], where we examined whether the participants can accurately perceive gaze direction of NAO robot and the resolution needed for the head angles of the robot to direct at different card locations on the board (see Fig. 4).

At the beginning, the cards were laid face down on the board, and then the player selected a card and tried to find a matching card. If the cards turned face up were similar (a pair of matching cards), then the player continued to match the cards; otherwise, the participant turned the cards face down and made a new trial/move. The goal was to find all pairs in the smallest number of tries/attempts and in shortest time possible. An attempt (try) consists of choosing two cards; the game ended when the participant found all the matching pairs. Although the game is better and more enjoyable when two players play against each other, it can also be played by a single player. The participant played the game alone, in the presence of either a robot or a human tutor.

**Fig. 2:**
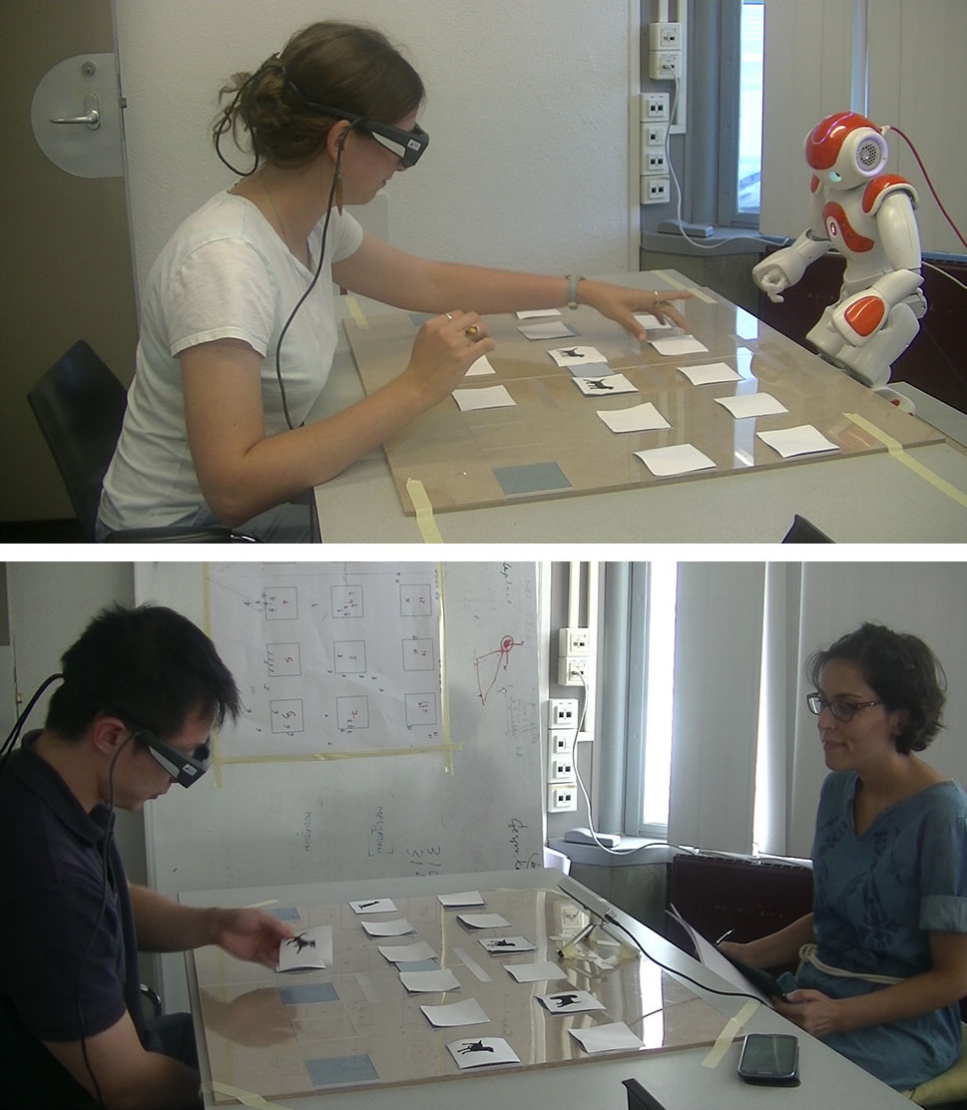
The set-up: human–robot set-up (top); human–human set-up (bottom)

#### Human-Human Interaction Setup

**Fig. 3:**
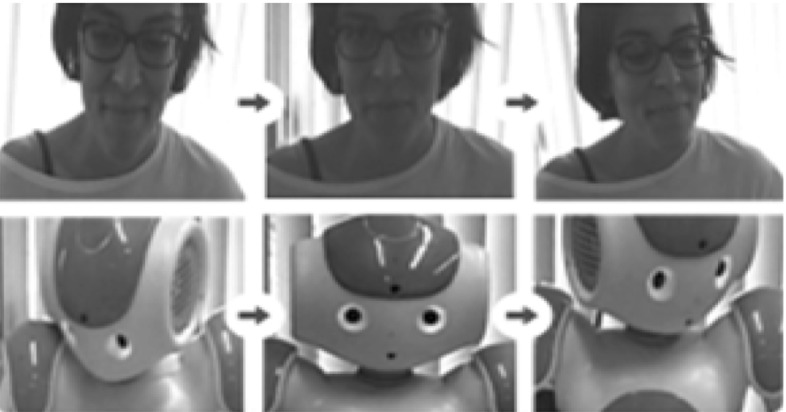
Tutor gaze captured from eye tracking videos: human tutor (top); robot tutor (bottom); tutor looks at the chosen card; looks to the participant, and then looks to matching card

Figure 2 (bottom) depicts the human–human set-up. The tutor and the participant sat across the table, approximately 160 cm apart. The human tutor was trained to follow a pre-defined protocol of steps that detailed the rules of how to introduce the game and the sequence of how to shift her gaze during the game. Figure 3 captures the sequence of tutors’ gaze (human and robot tutor) from the eye tracking videos in the help condition. The tutor first looks at the chosen card; then looks to the face of the participant, and then looks to the matching card. This sequence of shifts in gaze direction was consistent for both tutors (human or robot) in the help condition.

**Fig. 4:**
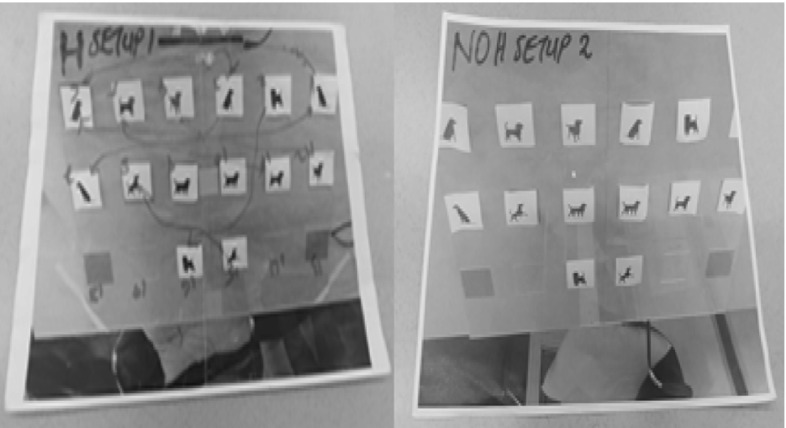
Card arrangement configurations for both conditions: set-up one (Help) left; set-up two; (No_Help) right

The same person acted as the tutor for all the participants, and during all sessions. In front of the tutor was a printed photo of the card locations on the board layout (see Fig. 4, left). After each session with a participant, the cards were re-arranged. In this set-up, we logged the gaze of both the tutor and the participant. The gaze data of the tutor were registered using the Eye-tribe gaze direction tracker, while the participant wore SMI eye-tracking glasses to capture their eye gaze behavior. The experiment was recorded using I-view ETG software, and also with a video camera. The reason for recording the gaze of the tutor was to examine if the tutor gaze was consistent in all sessions, and if varying behavior on the part of the tutor influenced the participant gaze data in any way.

#### Human–Robot Interaction Setup

In the robot condition, a humanoid robot NAO developed by Aldebaran Robotics [34], a personal computer, a web-cam, and the same memory game (see Fig. 2) was used. NAO is a 57 cm tall robot with a moveable head and facial features that bear a resemblance to those of a child. As a result of its minimalistic design and perception capabilities, NAO robot has been adopted widely for research focused on therapeutic training, or for general educational/pedagogical purposes. To develop the game, each card was labelled with a unique card code, and placed in a fixed position on the board layout marked with a head pitch and yaw angle on the computer layout (See Fig. 4 left). The algorithm was applied such that, the robot head angles shifted to the card position of the chosen card code, then to the face of the participant (assumed at NAO initial position), and then to the location of the matching card. The design of the help state gaze for the robot follows the concept of attention-directing gaze movement in human communication. Another aspect we considered is the timing of gaze behaviour; prior to conducting the experiment we invited fellow students to the lab and played the robot gaze motions to different cards on the table for them with two different timings. We used the timing of head movements that was regarded as more natural.

Figure 3 shows the image sequence of the designed tutor gaze

### Experimental Design and Conditions

The study was a two-by-two (Tutor_Type: Human or Robot) and (Help_Type: Help vs. No_Help) mixed factorial design experiment. The Tutor_Type variable was manipulated as a between-participants and the Help-Type as a within-participants. In the Help condition, the tutor provided gaze hints to help the participant find the matching cards. While introducing the game, the tutor informed the participant that she would help them. However, the tutor did not explicitly reveal the modality she would use to help. The tutor remained silent the entire session; when the participant turned the card upwards, the tutor gazed at the flipped card, looked to the participant and then to the matching card. In the No_Help condition the tutor remained silent and only stared at the participant. Participants were evenly assigned to play in the presence of either the robot or the human tutor. Each participant interacted with the tutor in both conditions of Help and No_Help, and the order of conditions was counterbalanced across trials. In both set-ups, the robot and the human tutors performed similar actions.

### Hypothesis

We formulated two hypotheses as outlined below regarding how help (presence of gaze hints) and the type of tutor (human/robot) would affect participants’ performance of the task, and also how the gaze of the participants would differ between interactions with the human and humanoid tutor.

- **Hypothesis One (H1)** Participants will perform better in the Help condition than in the No_Help condition.
  - **H1.1** Participants will complete the task in less time in the Help condition than in the No_Help condition.
  - **H1.2** Participants will complete the task with fewer tries in the Help condition than in the No_Help condition.
- **Hypothesis Two (H2)** The type of tutor (human/robot) will influence the participants’ task performance both in terms of time and numbers of tries.
- **Hypothesis Three (H3)** The type and the style of tutor will influence participants’ gaze behaviour with the tutor during the play
  - **H3.1** Participants will look more into the tutors’ face in the Help condition than in the No_Help condition.
  - **H3.2** Participants will look more into the robot tutors’ face than in the human tutors’ face.

### Measurements

To evaluate the above mentioned hypothesis we employed the following measures:

*Task performance* We identified two primary objective measures that are notably used to measure performance in memory game: (1) Duration:—the time it takes the participants to find all pairs of matching cards on the table; and (2) Number of tries:—the total number of attempts required to find all matching cards. A “try” consists of choosing two cards. All sessions were video-recorded to facilitate the analysis according to both measures.

*Gaze Behavior* While playing the game, the participants wore SMI eye tracking glasses, to capture the gaze direction. We recorded these data, in order to compare the gaze patterns when the participants interacted with the human and the robot. Eye tracking provides data with high temporal resolution and can, therefore, reveal detailed patterns of gazing behavior.

*Perceptions* We used a questionnaire to evaluate participants’ perceptions of the tutor behaviors, particularly perceived likeability, perceived presence of the tutor, and also the feedback about the task. We included open-ended questions in the questionnaire asking the subjects to list the helping cues that they observed or searched for in the tutors’ behavior. In the end, we conducted semi-structured interviews to assess whether the participants perceived any differences between the two conditions of the game.

### Procedure

Before the participants entered the room, the experimenter placed the cards in their correct locations on the board layout as shown in Fig. 4.

Upon receiving informed consent, the experimenter verbally provided the participant with details regarding the task and directions on how to play the game, including the use of SMI Eye-Tracking Glasses. At this point, the experimenter made sure not to disclose the objective of the study. The SMI eye-tracking glasses were fitted on the participant. The experimenter then performed a calibration procedure for the eye-tracking system.

The participants followed the tutors’ instructions to complete the game. Once the game ended, the participants filled out a post-experiment questionnaire. After each session, the study took a pause of around 4 min; the participant was requested to wait outside the room for the experimenter to reorganize the game for the second session. The same procedure was then repeated for the other condition. After interacting with the tutor in both conditions (Help and No_Help Condition), the experimenter collected the demographic details and interviewed the participants to get more information on any differences they may have observed in the tutors’ behavior between the two conditions.

### Participants Profile

The twenty participants who took part in the study (Gender: eleven males and nine females, Age: 19–33) were students recruited from the university campus. The participants were from different cultural backgrounds, namely, China and different European countries. The game sessions took approximately thirty minutes. Each participant received a coupon worth ten Euro at the end of the experiment for their participation.

## Results

The paragraphs below report on the results from performance, gaze and subjective analysis. We first describe results from the performance measures, in order to provide background information for the eye-tracking and subjective measures.

**Fig. 5:**
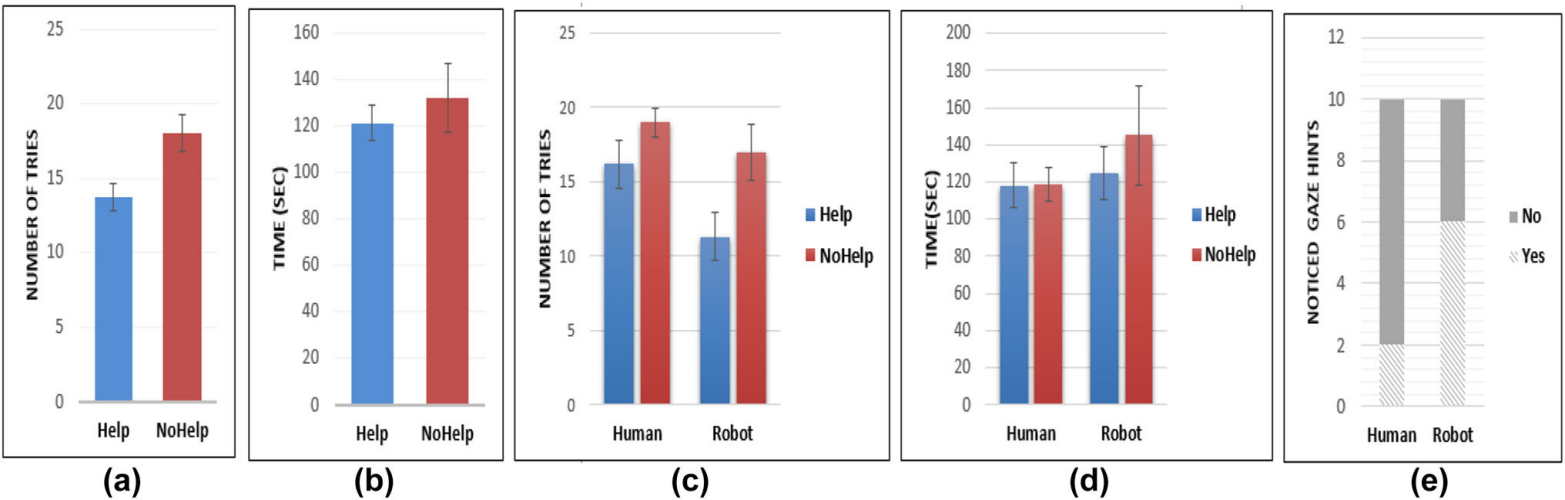
**a** Number of tries with and without help. **b** The time (seconds) it took participants to find matching cards with and without help. **c** The number of tries with and without the help for the two tutors. **d** The time (seconds) to match all the cards with and without the help from the two tutors. **e** The number of participants who noticed the gaze hints during help condition for the two tutors

### Performance Measures

For objective analysis, we conducted a mixed-model ANOVA in SPSS, with the repeated measure Help_Type (Help vs. No_Help) as the within-subject factor and the Tutor_Type (Robot or Human) as the between-subject factor. We analyzed the results of 20 participants (10 for the Robot tutor condition, and 10 for the Human tutor condition, for a total of 20 trials in the Help condition and 20 trials in the No_Help conditions across both tutor conditions. We analyzed the effect of Help and Tutor type on the following two performance measures:

*Duration* We obtained the duration from video recordings, this being the period between the participant starting to play the game (first selection) and completing it.

*Number of tries* We counted the number of tries/attempts that participants used from our video recordings.

Tables 1 and 2, and Fig. 5 provide results from the defined objective measures.

**Table 1:** Performance measures; duration (s) and number of tries

Tutor_Type	Help		No_Help		N
	Mean	SD	Mean	SD	
Durations (s)					
Human	118.00	38.306	118.70	45.019	10
Robot	124.40	27.774	145.10	83.523	10
Average	121.20	32.730	131.90	66.692	20
Number of tries					
Human	16.20	5.116	19.00	5.121	10
Robot	11.30	3.057	17.00	5.925	10
Average	13.75	4.811	18.00	5.487	20

**Table 2:** Effect of Help at different levels of the Tutor_Type

Tutor_Type	Help_Type		MD	SE	Sig.
Duration (s)					
Human	Help	No_Help	$-$ 0.7	23.13	0.976
Robot	Help	No_Help	$-$ 20.7	23.13	0.383
Number of tries					
Human	Help	No_Help	$-$ 2.8	2.27	0.233
Robot	Help	No_Help	$-$ 5.7	2.27	${0.022}^*$

*statistical significance, $$P < 0.05$$

**Effect of Tutor_Type on performance measures** Tests of between-subject effects based on the F tests of the averaged variables show a significant multivariate effect of Tutor_Type on performance measures ($p=0.048$). There was no significant main effect of Tutor_Type on duration ($F (1, 18) =0.913, p=0.352$). However, we found a significant main effect of Tutor_Type on the number of tries ($F (1, 18) =5.253, p=0.034$).

**Effect of Help_Type on performance measures** Tests of within-subject effects based on the F tests of the averaged variables shows a significant multivariate effect of Help_Type ($p=0.003$). There was a significant main effect of Help_Type on the number of tries ($\hbox {F} (1, 18) =7.009, p=0.016$). However, there was no significant main effects on duration between the Help and No_Help conditions. ($\hbox {F} (1, 18) =0.428, p=0.521$).

We found no significant Help_Type by Tutor_Type interaction ($p = 0.654$). Similarly, there was no Significant Help_Type by Tutor_Type interaction effects on the duration F $(1, 18) =0.374, p=0.549$ and number of tries $(\hbox {F} (1, 18) =816, p=0.378$).

**Effect of Help at different levels of the Tutor_Type**

*Duration* For the Human condition, there was no significant difference in duration of the Help and No_Help condition ($p=0. 976$). Similarly, the mean difference in duration between the Help and No_Help condition was not significant for the Robot condition ($p=0. 383$).

*Number of tries* For the Human condition, there was no significant difference in the number of tries between the Help and No_Help ($p =0. 233$). However, for the Robot condition, the mean difference in the number of tries between the Help and No_Help condition is ($\hbox {MD} = 5.7$), was significant ($p=0.022$); indicating participants performed significantly better, with fewer tries, with help from the robot tutor than without help.

*Counterbalanced orders: performance measures*

Participants interacted with the tutor in both conditions (Help and No_Help) in a different order: Group 1 (Order: Help; No_Help) and Group 2 (order No_Help; Help). To examine order effects on performance measures, we created a data matrix with six columns. Subject ID, Group: Group 1 (Help; No_Help) and Group 2 (No_Help; duration and number of tries, two for each condition. From the analysis, we found a significant Group * Help interaction effect on duration; thus, there was an overall order effect. The differences were larger for Group 2 (No_Help; Help) than for Group 1 (Help; No_Help). However, we found no significant Group * Help interaction effects on the Number of tries.

Pairwise comparisons (adjustment for multiple comparisons: Bonferroni) between the Robot and the Human condition, reveal that participants used significantly fewer tries with help from the Robot tutor than with the help from Human tutor ($p=0.018$, two-tailed). However, there were no significant differences in the number of tries for the two groups (Human and Robot) when help was not present ($p=0. 430$, two-tailed), assuming equal variances. Comparing both groups on duration, we found no significant differences in both Help ($p=0. 674$, two-tailed) and No_Help ($p=0. 391$, two-tailed) conditions.

**Fig. 6:**
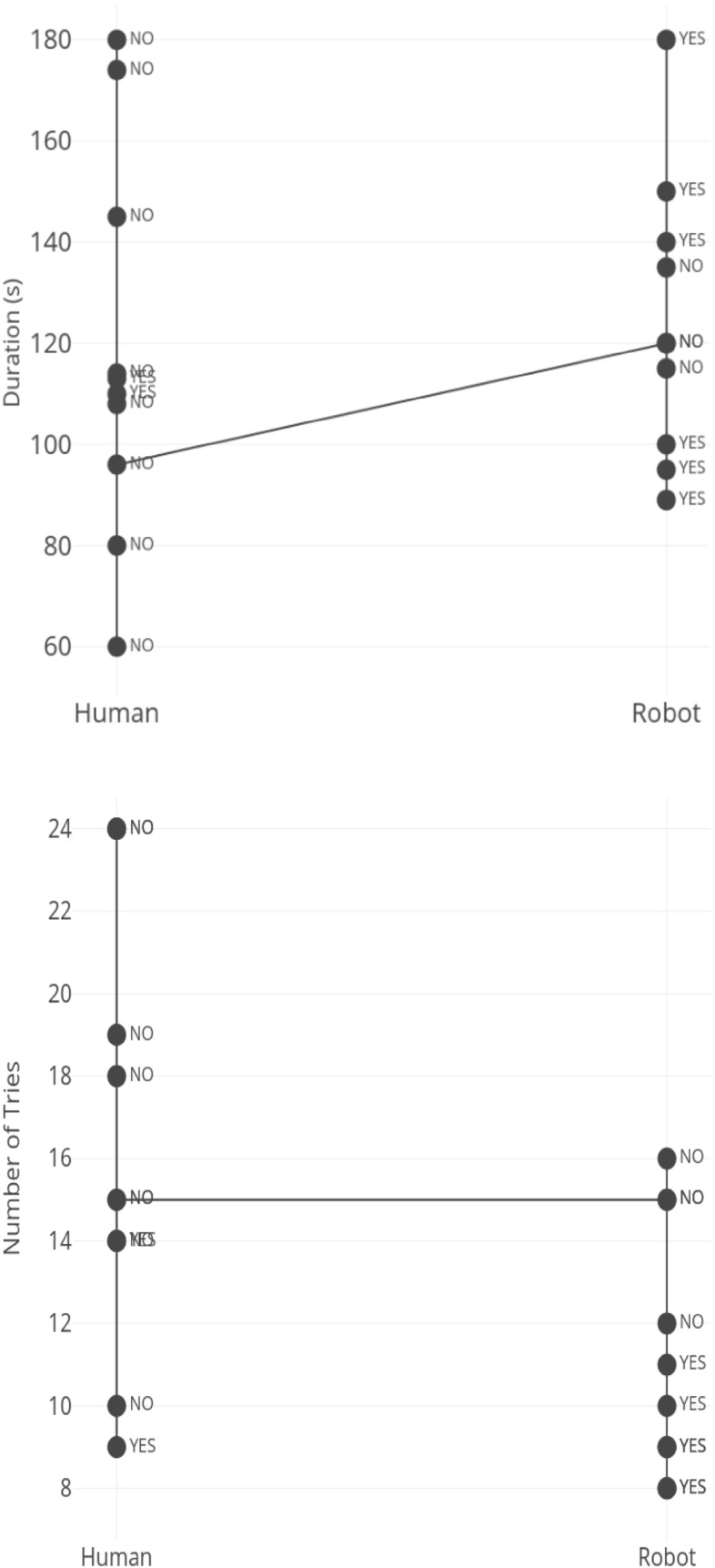
Noticing gaze in help condition and performance measures (top; duration; bottom; number of tries)

Twenty percent of the participants in the Human condition reported noticing help gaze hints while sixty percent in the Robot condition said they recognized gaze hints. For the Human condition, there was no significant difference between those who reported identifying the gaze hints and those who did not report identifying the gaze hints across both measures (duration and number of tries). For the Robot condition, there was a significant correlation between noticing gaze hints and the number of tries. Participants who reported seeing the gaze hints in the Robot condition performed significantly better, measured by the number of tries than those who did not report identifying the gaze cues. However, there was no significant difference in duration for those who identified gaze hints in the robot condition and those who did not report identifying the gaze hints. We provide probable explanations for these findings in the discussion section. Figure 6 shows how individuals who did not recognize that the tutor was helping performed if compared with participants who made use of the hints. The figure shows the number of attempts and duration in the “Help” condition with text representing subjects’ awareness of the hints (YES or NO) for both Human and Robot Tutors.

### Eye-Gaze Measures

**Fig. 7:**
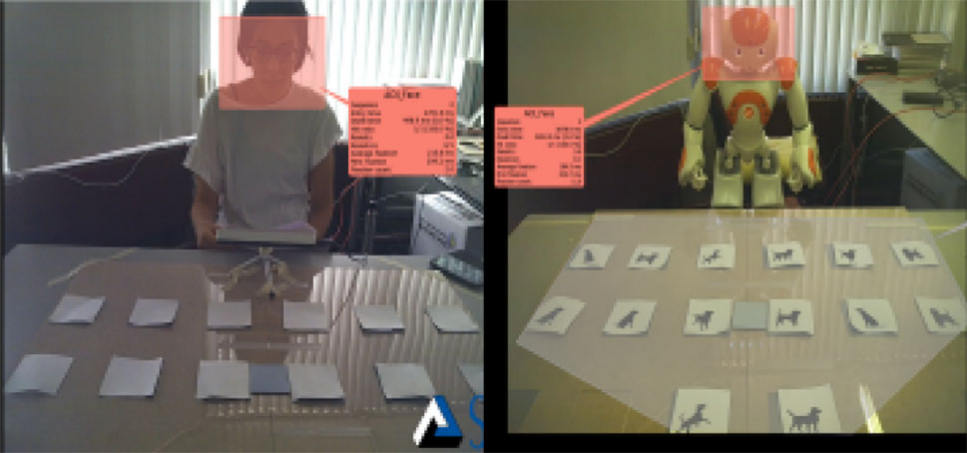
Area of interest (AOI) region: face of the robot and the human tutor

In this subsection, we analyze the eye gaze patterns when the participants interacted with the human and the robot tutor. To analyze the recorded gaze data from the video, we used Begaze software to create custom trials of the video recordings, which included only the segment from the participant starting to play the game until game completion. From the trial images, we cropped the face of the robot and that of the human tutor as an area of reference (AOI_Face) as shown in Fig. 7 below.

Next, we exported the metrics related to the trials and area of interest to SPSS software. We analyzed the effect of Help and Tutor type on the following eye gaze measures:

*Fixation count* this refers to the number of fixations within an area of interest(AOI_face).

*Fixation time* this refers to the total fixation time within an area of interest (AOI_Face) and is often associated with attention and visual processing.

*Dwell time* The time spent looking at the area of interest, calculated by summing up the time the gaze coordinates were within the face area (AOI_Face) (Table 3).

**Table 3:** Eye-tracking measures

	Human	Robot	Average
Fixation count (AOI_Face)			
Help	4.6	19.8	12.2
No_Help	2.9	5.6	4.2
Average	3.7	12.7	8.2
Fixation time (Ms.) (AOI_Face)			
Help	1097.9	4205.7	2651.8
No_Help	558.2	1001.5	779.9
Average	828.1	2603.6	1715.9
Dwell Time (Ms.) (AOI_Face)			
Help	1760.53	5749.22	3754.88
No_Help	285.76	4237.06	2262.41
Average	1028.15	2408.14	3008.65

There was no significant main effect of Help on Fixation count: $p=0.081$; Fixation time: $p=0.070$. However we found that there was a significant main effect of the tutor type on the fixation measures: Fixation count: $p=0.020^*$ Fixation time: $p=0.050^*$.

Comparing the effect of Help on eye-tracking measures at different levels of the tutor, we found significant differences in both Help and No_Help conditions for the robot condition for both Fixation measures; Fixation_count $(p=0.028)$and Fixation time $(p=0.028)$. However, there were no significant differences on both measures for the human condition during Help and the No_Help conditions. Results show that the total number of eye fixations on the robot face was significantly higher than the number of those on the human face.

### Participants’ Perceptions

**Fig. 8:**
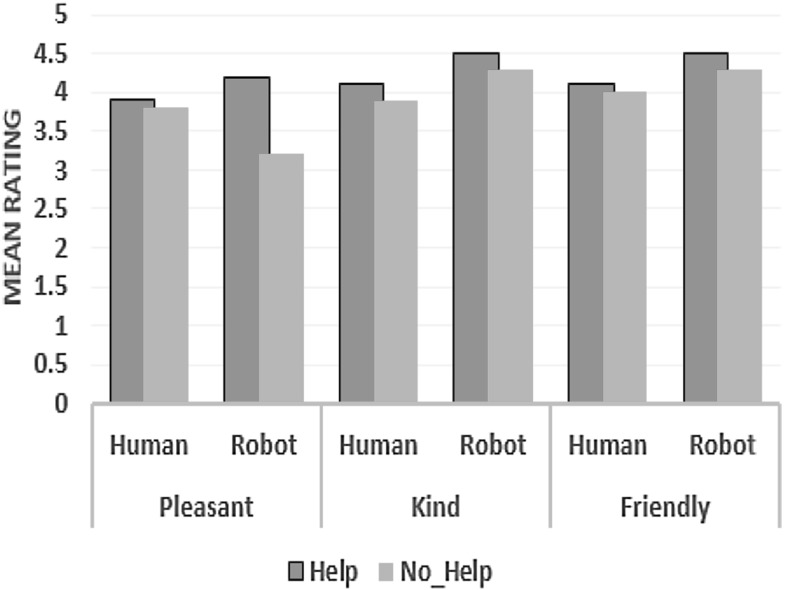
Comparing the effect of help on likeability measures at different levels of the Tutor_Type

For subjective analysis, we conducted a mixed model analysis of variance in SPSS, with the repeated measure Help_Type (Help vs. No_Help) as the within-subject factor and the Tutor_Type (Robot or Human) as the between-subject factor. To facilitate the analysis of the Likert scale data, we coded the data as follows: 1: Completely Disagree; 2: Disagree; 3: Neutral; 4: Agree; and 5: Completely Agree. We also included the ‘not applicable option, and these data were treated as missing values during the analysis. Figures 7 and 8 summarize the results from our subjective measures, particularly the perceived likeability and presence of the tutor.

*Likeability* Regarding the aspect of tutors’ likeability, participants were asked to rate the following statements about the tutor: Tutor was pleasant; Tutor was kind; and Tutor was friendly in a five-point Likert scale.

There was a significant multivariate effect of Help on Likeability measures $(p=0.046)$. We found significant main effects of Help on the Pleasant measure $[F (1, 16) =7. 828, p=0. 015]$. However, there was no significant effect of Help on the participants rating of tutors’ Kindness F $(1, 16) =1. 361, p=0. 260)$, Friendliness $(\hbox {F} (1, 16) =2. 028, p=0. 174)$. There were no significant main effects of Tutor_ type on any of the Likeability variables used. We found no significant multivariate interaction (Tutor_Type * Help_Type) effects on Likeability measures $(p=0.144)$. We found significant interaction effects interaction on the Pleasant measure $(\hbox {F} (1, 18) =4.893, p=0.040)$; However, there were no significant interaction effects on the other measures.

Pairwise comparisons between the two tutors on the Likeability measures showed no significant difference regarding how participants rated both tutors for all likeability measures used.

Comparing the effect of Help_Type on Likeability measures at different levels of the Tutor_Type, we found that the participants rated the robot tutor as more socially Pleasant $(p=0.005)$ in Help condition. However, there was no significant difference for Kindness and Friendliness. On the other hand, there was no significant difference in how participants rated the human tutor in both Help and No_Help conditions across all Likeability measures used.

*Presence* A test of within-subject effect shows a significant multivariate effect of Help on presence measures $(p=0.003)$. There was significant main effects of Help_Type on Tutor_Presence $(\hbox {F} (1, 18) =15.059, p=0. 001)$; Tutor caught the participants attention F $(1, 18) =9.529, p=0. 006;$ and Tutor was attentive $F (1, 18) =6.600, p=0.019))$. A test of between-subject effect shows a significant multivariate effect of Tutor_Type on Presence measures ($p=0. 001$). There was a significant main effect of Tutor_Type on Tutor caught the participant’s attention measure F $(1, 18) =5.921, p=0. 026)$; However the main effects of Tutor_Type on Tutor_Presence $(\hbox {F} (1, 18) =3.009, p=0. 100)$; and Tutor was attentive $\hbox {F} (1, 18) =1.638, p=217)$ were not significant.

We found no significant multivariate interaction (Tutor_Type* Help_Type) effects on Presence measures $(p=0.090)$. We found significant interaction effects interaction on Tutors’ perceived presence ($\hbox {F} (1, 18) =4.366, p=0.051$); and how the Tutors’ behaviour caught the attention of the participant [F $(1, 18) =4.235, p=0.054$]; but the interactions effects on Tutors’ perceived attentiveness [F $(1, 18) =0.492, p=0.491$] were not significant. Pairwise comparisons on the effect of Help on Presence measures, at different levels of the Tutor_Type, show that participants rated the robot tutor as more socially present $(p=0.001)$, and more attentive $(p=0.33)$ in the Help condition than in the No_Help condition. Moreover, they indicated that the robot tutor behavior caught their attention more during help than when there was no help (p = 0.002). However, we found no significant difference on how the participant rated the human tutor across all presence measures in both Help and No_Help conditions.

**Fig. 9:**
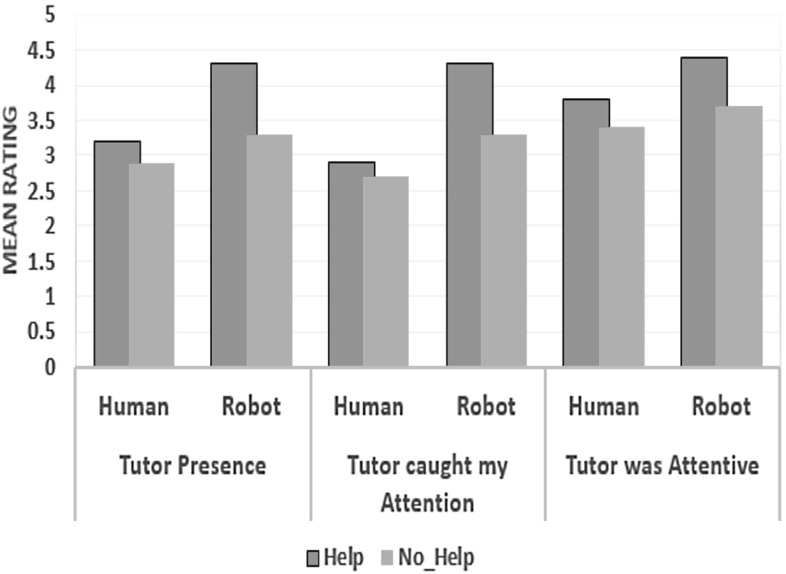
Comparing the effect of help on presence measures at different levels of the Tutor_Type

Pairwise comparisons based on the estimated marginal means between the two tutors on the presence measures show that the robot tutor behavior caught the attention of the participants significantly more compared to the human tutor $(p=0.026)$. However, there was no significant mean difference in how participants rated both tutors for the other two measures difference in how both tutors were rated for the other two measures (Fig. 9).

### Post-Experiment Interview

In the end, we conducted a post-experiment interview. We asked participants whether they noticed the help from the tutor and, if they did, whether that influenced their choice of cards. We also asked what cues they expected the tutor would use to help them in the game. Lastly, we examined how they perceived the robot tutor head movements: whether they were natural, too fast, or too slow, and if they considered the behavior of the robot tutor to be automatic.

Eight (8) participants in the human condition said they did not notice the help, i.e., the gaze hints from the tutor. Most of them indicated they were focused on the game and did not look at the tutor. At least eighteen (18) of all the twenty (20) participants in both human and robot conditions said they expected verbal/vocal/audio help from the tutor, as illustrated in the excerpts below:

P003: [Human condition] “I expected verbal hints from the tutor, I didn’t pay attention to the tutor.”
P006: [Human condition] “I noticed the gaze hint but it took a while to get to the eyes. I expected speech, for example, when I get closer to the matching card the tutor says something like warmer!!, or hey, you have seen this card before. If there is no help, you are forced to remember, but now I relied on help.”
P009: [Human condition] “I was focused on the game so I did not look at the tutor, maybe in the future you can put lights with different colors under the card or give some sound instructions. Audio feedback could be important”.
P017: [Robot condition] “I noticed and used the help as direction, but I prefer speech.”

Four (4) people in the robot group did not notice the gaze hints. However, all of them reported seeing the head movements, but felt like the robot was following their moves rather than directing their attention to the matching cards as illustrated in the following excerpts:

P016: [Robot condition] “I noticed the robot was looking around, but it felt like it was following my moves, not showing me the positions. I felt as if, we were both looking.”
P018: [Robot condition] “I did not get help! It seemed like he sees what I see! I was more focused on the task, and I thought he was watching what I was doing.”
P011: [Robot condition] “I was focusing on the cards, and not looking at the robot. I thought the help would be vocal; the robot looked like it was moving with me, following me.”
P012: [Robot condition] “I did not feel like it was helping, just thought he was looking at the cards I was turning. I did not get the hint.”

We recorded mixed responses from participants as regards how they perceived the direction of gaze for the robot tutor.

P018: [Robot condition] “I got that the robot was helping, it was looking at the right card. Later, I felt the movements of the head were slower.”
P020: [Robot condition] “I got the tutor help—it looked at my card, then me, and then to the matching card. I just got one problem—to read the gaze direction from the angle. The head movements were pretty well-paced, not too slow, not too fast.”
P14: [Robot condition] “I got that the tutor was helping, was looking at the right card, but it was not easy to tell which one it was looking at. The robot felt natural and the speed was nice. I felt like the robot was fully automatic.”
P16: [Robot condition] “Yes, I noticed the help, pretty natural, difficult to see the direction, I was not sure which card when the cards were closer.”

## Discussion and Conclusion

This paper presents an experimental study designed to examine whether gaze cues from a human or a robot tutor can direct attention and influence the choices of human partners in a card matching game. Specifically, users are asked to pick matching card pairs and have varying levels of assistance in locating the matching card. The conditions of assistance are Help and No_Help from either a human tutor or a robot tutor. The help involves the tutor looking at the matching card once the player has made a card selection. The paper details a study design on comparing task performance measured by time and number of tries of Help versus No_Help, and robot versus human tutoring.

Findings from the study support the formulated hypotheses. In our first hypothesis (Hypothesis One), we projected that participants would perform better in the Help condition than in the No_Help condition. By measuring the number of tries needed to complete the game, we found that the participants used significantly fewer tries to find all the matching cards with help from the tutor than without help. However, we found no significant difference between the duration in the two conditions. In our second hypothesis, (Hypothesis Two), which predicted that the type of tutor (human/robot) would influence the participants’ task performance both regarding time and numbers of tries. According to participants awareness of the tutors’ hints, a significantly higher number of participants reported identifying the help cues and using the gaze information to pick the matching cards during the Robot-Help condition than in the Human-Help condition. Consequently, participants performed significantly better, measured by the number of tries, with help from the robot tutor than from the human tutor. We found that participants identified all the pairs of matching cards with significantly fewer tries with help from the robot tutor $(p=0.022)$ than without help. However, there was no significant difference in the number of tries, with or without help, in the human tutor condition $(p=0.233)$.

Further analysis shows a significant correlation between noticing gaze hints and the number of tries/attempts. Participants who reported seeing the gaze hints in the robot condition performed significantly better, measured by the number of tries than those who did not report noticing the gaze cues. However, there was no significant difference in duration for those who identified gaze hints in the robot condition and those who did not report identifying the gaze hints. For the human condition, there was no significant difference between those who reported identifying the gaze hints and those who did not identify the gaze hints across both measures. However, the few participants who noticed the help in the human condition noticed it too late in the game to take advantage of it.

The significant difference between the number of participants who noticed the gaze hints of the human or the robot tutor might relate to diverse factors. First, the novelty effect of the robot increased participants’ attention, supported by the fact that participants with the robot tutor spent more time to fulfill the task even when the number of tries was smaller. Analysis of the eye gaze data indicated that participants spent more time looking at the robot hence they needed more time to complete the task. Another explanation could be that the sounds of the robot’s motors displaying the head movements—to perform gaze behavior—possibly attracted participants attention, making the robot’s gaze behavior more salient than human’s. Besides, the robot’s gaze behavior is achieved with large head movements, whereas the human tutors’ gazes are much subtle, based more on the eyes movements than in the head motion. According to the particular salience in the experimental setting, we can consider that robots’ gaze behavior is overt while that of the human is a covert cue, which affects its communicative effectiveness in the context of assisting the player. Though covert cues can influence interaction even without being aware of them, in this particular context and regarding the game flow, the hints providing information about the matching card position should be noticed by participants to be effective.

With this respect, our findings differ from Mutlu et al. [28] or in collaborative scenarios (Palinko et al. [31]) who showed that gaze is a powerful communicative signal without explicit awareness from the observer. In the current study, the lack of awareness overrides the informative content of gazing, which we believe is because of the lack of subtlety of the NAO eyes which do not have the impactful embodiment of human-like eyes. Moreover, in the human condition the concept of intimacy regulation, according to which humans control their gazes to regulate the level of intimacy with their interaction partners [4] also applies. We conjecture that in the human condition participants restricted the gaze behavior according to social rules, which was not present in the robot case. Additionally, in the human condition, subjective evaluation indicated that a majority of participants expected verbal help from the tutor, as the more natural modality of communication in the face to face situation, even more provided the human tutor addressed the participant verbally in the introduction of the activity.

Results do not show a significant effect on game duration, even during the robot condition. There are several possible explanations for this fact. Firstly, as soon as the participant noticed that the robot tutor was helping, they waited until the robot gazed at the matching card, even when they had an idea of where the matching card was. Secondly, as revealed in the interview responses, it took a while for some of the participants to read the gaze direction of the robot. The difficulty in reading gaze could be attributed to the limitations of the robot used as the experimental platform for this study, as it lacks articulated eyes. The third probable reason is the duration of head motions during attention shifts from the flipped card to the face of the participant, and then to the matching card. Again, as shown in the qualitative responses, some of the participants indicated that the head movements of the robot tutor were slow. In this sense, the robot’s help could be considered detrimental regarding task performance only when measured by time: waiting for robots’ hints increased the execution time while improved the accuracy of selections.

We recorded diverse responses from participants as regards how they perceived the direction, timing and intent of head movement (gaze) of the robot tutor. A few of the participants reported identifying the robot head movements but felt like it was following their tries rather than directing their attention to the matching card, missing the informative content of tutors’ gaze. This brings up the issue of the timing of gaze behavior and directions of head movements, which are two interesting aspects of our future work. Further analysis of the gaze behavior of the participants collected during the experiment will reveal finer, micro-level details of the interactive player-tutor looking behavior, including complex gaze patterns (i.e. gaze following, joint attention) to gain an understanding of the differences between human–robot and human–human nonverbal communication. Interesting coordinated sequences to investigate are responsive gaze behaviors to either the robot or the human tutor head turn or gaze shift. Besides, the analysis of sequences of coordinated gaze behavior could shed light on the participant’s attributions of tutors’ intent in the flow of the game. The attributed meaning of tutor behavior could be inferred from the moment when participants check tutor gaze behavior—look at the face-, for instance before selecting a card—looking for cues- or just immediately after a card selection—looking for confirmation.

Finally, we observed that participants in the robot condition appeared unperturbed, however, in the human condition, participants seemed a little uneasy. This observation, and especially the reasons for it can be investigated in future studies. One possible explanation is the unnaturalness of the human tutor interactive behavior, shifting from a casual verbal based communication during the introduction and the briefing phase into an absence of verbal communication during the game in both Help and No_Help conditions.

The use of a card game aims to add to our research line on social inclusion which includes training social skills to children with ASD [5, 19], emotional bonding [15] and improving the involvement in activities of elderly with dementia [32] which was shown to be very beneficial for these user groups. In such settings often games are used to encourage the children to better engage in training practices [5, 19, 24]. Huskens et al. [19], and Barakova et al. [5] have incorporated game-based training in clinical studies, while Palinko et al. [31] gives a plausible framework for introducing robots with designed gaze behaviors in general education. Perugia et al. [32] used cognitive games and robots to engage elderly by mental stimulation on an emotional level.

Future work involves examining the temporal aspects of gaze and movements in human–human interactions to build more realistic interactive robot gaze behaviors. In the future, we hope to combine robot’s head-directions behaviors, different gaze movements, and other social cues derived from Time and Flow Effort of Laban Movement Analysis [25]. Additionally, to improve our design and communication capabilities, we will combine NAO with a gaze tracker, to detect gaze directions of the human and to control robot responses.
